# Volumetric Absorptive Microsampling in Toxicology

**DOI:** 10.3390/toxics13010025

**Published:** 2024-12-30

**Authors:** Bruno Pires, Gonçalo Catarro, Sofia Soares, Joana Gonçalves, Tiago Rosado, Mário Barroso, André R. T. S. Araujo, Eugenia Gallardo

**Affiliations:** 1 Centro de Investigação em Ciências da Saúde (CICS-UBI), Universidade da Beira Interior, Av. Infante D. Henrique, 6200-506 Covilhã, Portugal; bruno.mg.pires@gmail.com (B.P.); goncalo.catarro@hotmail.com (G.C.); sofia_soares_26@hotmail.com (S.S.); joanadgoncalves13@gmail.com (J.G.); tiago.rosado@ubi.pt (T.R.); 2 Laboratório de Fármaco-Toxicologia, UBIMedical, Universidade da Beira Interior, Estrada Municipal 506, 6200-284 Covilhã, Portugal; 3 RISE-Health, Departamento de Ciências Médicas, Faculdade de Ciências da Saúde, Universidade da Beira Interior, Av. Infante D. Henrique, 6200-506 Covilhã, Portugal; 4 CERNAS-IPV Research Centre, Polytechnic University of Viseu, 3504-510 Viseu, Portugal; 5 Grupo de Investigação Sobre Problemas Relacionados Com Toxicofilias, Centro Académico Clínico das Beiras (CACB), Universidade da Beira Interior, Av. Infante D. Henrique, 6200-506 Covilhã, Portugal; 6 AlphaBiolabs, 14 Webster Court, Carina Park, Warrington WA5 8WD, UK; mbarroso@alphabiolabs.com; 7 Serviço de Química e Toxicologia Forenses, Instituto de Medicina Legal e Ciências Forenses—Delegação do Sul, 1169-201 Lisboa, Portugal; 8 BRIDGES—Investigação Biotecnológica para a Inovação e Design de Produtos de Saúde, Instituto Politécnico da Guarda, Avenida Dr. Francisco Sá Carneiro, n.º 50, 6300-559 Guarda, Portugal; 9 LAQV, REQUIMTE, Laboratory of Applied Chemistry, Department of Chemical Sciences, Faculty of Pharmacy, Porto University, Rua Jorge Viterbo Ferreira, No. 228, 4050-313 Porto, Portugal

**Keywords:** volumetric absorptive microsampling, clinical toxicology, forensic toxicology

## Abstract

Volumetric absorptive microsampling (VAMS) is an emerging technique in clinical and forensic toxicology. It is recognized as a promising alternative to traditional sampling methods, offering an accurate and minimally invasive means of collecting small volumes of biological samples, such as blood, urine, and saliva. Unlike conventional methods, VAMS provides advantages in terms of sample stability, storage, and transportation, as it enables samples to be collected outside laboratory environments without requiring refrigeration. This review explores several VAMS methodologies, with a particular focus on its application for the quantification of drugs and other substances in clinical and forensic toxicology. It compares VAMS to other microsampling techniques, such as dried blood spots (DBSs), highlighting VAMS’s superiority in addressing issues related to sample volume consistency and environmental impact. Despite its advantages, VAMS also presents certain limitations, including higher costs and difficulties in detecting underfilled samples. Overall, VAMS stands out as a microsampling technique with the potential to enhance patient compliance and operational efficiency, positioning itself as a viable tool for toxicological analysis in both clinical and forensic contexts.

## 1. Introduction

**Figure 1:**
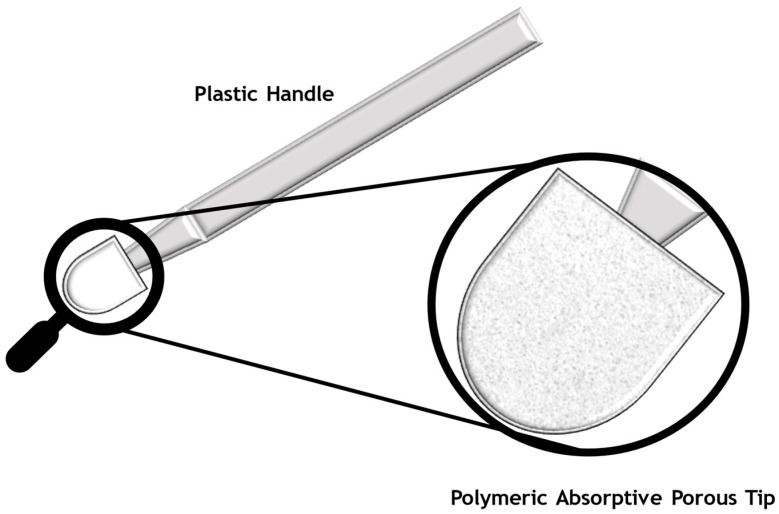
The VAMS device used to sample a biological specimen.

The accurate quantification of drugs in biological specimens is of utmost importance for preclinical and clinical studies, therapeutic drug monitoring (TDM), diagnostics, and forensic toxicology analysis [1,2]. In recent years, novel sampling techniques with reduced sample volumes have been emerging in order to overcome drawbacks presented by gold-standard sampling techniques such as venipuncture [3,4]. Volumetric absorptive microsampling, more commonly referred to as VAMS, is a miniaturized sampling technique that allows for the simple, accurate, and non-invasive collection of pre-determined amounts of whole blood, urine, plasma, oral fluid, or any other biological liquid matrices, although blood was the first biological sample for which this technique was developed [5]. VAMS utilizes a device comprising a plastic handle with a small absorptive polymeric tip, allowing for the absorption of a fixed amount of volume through direct contact with the desired fluid matrix [5,6]. The polymeric porous material used for the tip is hydrophilic, with a diameter of about 4 mm that can absorb 10 µL, 20 µL, or 30 µL of sample, depending on the tip size (Figure 1) [7,8]. The VAMS handle is designed in a way that prevents accidental contact between the sampler tip and surfaces during storage and shipping. Just like the dried blood spot (DBS) approach, VAMS tips present the advantage of permitting the storage and shipping at room temperature [3]. Mitra^®^ are devices based on the patented VAMS^®^ technology (Neoteryx, Torrance, CA, USA); however, these devices are plastic-based manufactured devices, and only collect one type of sample per device, culminating in a great environmental impact due to the large quantity of wasted plastics [9,10]. The devices’ shelf-life is 1.5 years, and their transport and storage are facilitated by their reduced size and the fact of being portable [8]. Also, they can be posted by regular mail, and not as a biohazard or in a refrigerated state, having the advantage of room-temperature storage without compromising their sample stability [9,10].

**Figure 2:**
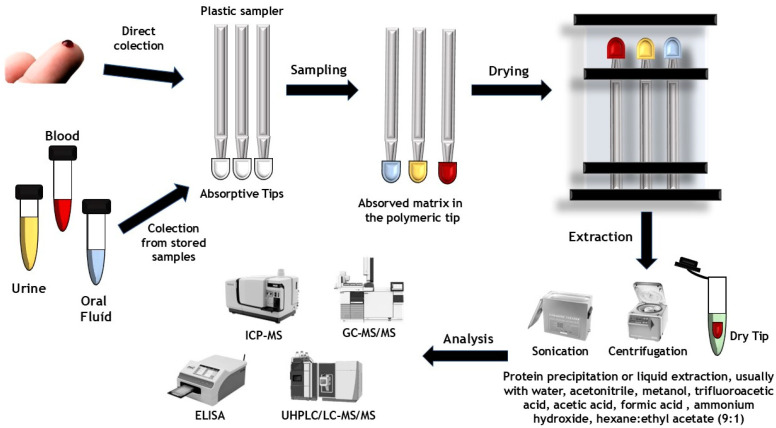
VAMS sampling and procedure representation.

Due to its absorption mechanism, VAMS can overcome the necessity of using micropipettes and any other volume-sampling devices that may or may not be user-dependent, eliminating potential pipetting errors and facilitating the collection process [7,8,11,12]. It also allows the collection of whole blood samples without resorting to the extremely invasive procedure that is whole blood collection via classical venipuncture [7,8,11,12]. Due to the small volumes required and the fact that a single drop of blood coming from a harmless “finger prick” can be quickly absorbed into the polymeric tip, VAMS became a very attractive technique, with an ability to be used outside of a laboratory environment, in a controlled and easy faction [7,8,11,12]. Practically, anyone can self-collect high-quality blood microsamples while using a VAMS device. The VAMS device contains vented cartridges where the samples can be safely stored for drying and sent for laboratory analysis [7,8,11,12]. A simplified version of the whole VAMS procedure can be seen in Figure 2. The sample collection is made through the direct contact of the tip with the fluid matrix, allowing it to be absorbed in a few seconds [8]. In the case of blood samples collected by “finger prick”, it is recommended to clean the fingertip before pricking [3,8]. The first blood drops are usually discarded to avoid contamination from skin, fibers, or alcohol residues, and then, subsequent droplets are sampled [3,8]. During sample collection, the tip is filled by holding the handle at an angle of 45° and dipping only the tip into a drop of blood [3,8]. The tip of the sampler should not be completely plunged into the blood sample since this may cause overfilling [3,8]. After the absorption is complete, the VAMS kit is placed to dry for a determined set of time [8]. The compounds present in the dried sample are later extracted under optimized conditions and analyzed, most commonly, through liquid chromatography [8].

The emergence of VAMS is justified by overcoming some disadvantages observed in conventional techniques, as well as for alternative extraction techniques such as dried matrix spots (DMS), plasma extraction cards, microneedle, etc. [3,13,14,15,16]. Table 1 lists each microsampling technique’s typical applications, benefits, and drawbacks.

DMS, which is the most similar to VAMS, uses a microsampling approach to collect liquid biological matrices like saliva (dry saliva spots, or DSS) or blood (dried blood spots, or DBS) while also eliminating sample-freezing steps and minimizing enzymatic degradation [3].

However, the main obstacle in this microsampling approach is the effect of hematocrit. The hematocrit presents inter-individual variability, leading to different blood viscosities in each individual and, consequently, differences when blood is applied and spread on the card, as well as the volume of sample collected [3,17]. Higher hematocrits lead to smaller and more concentrated spots on the paper, leading to a bias in the results obtained [3]. In samples with high hematocrits, there may also be difficulties in the desorption of the analytes, as the red blood cells can be retained on the card [17]. Since the hematocrit relates to the proportion of red blood cells, potassium that is predominantly located intracellularly has been used as a suitable marker to estimate this parameter [3,17]. In dried blood sample analysis, the hematocrit tends to influence recovery, so it is pertinent to determine its value as a way to verify if it is within the validated range [8,11,12]. Additionally, selectivity, stability, and recovery can also be affected if the matrix is aged [3,18]. According to the literature, with the VAMS technique, no significant differences are observed in aged samples or matrix effect [19,20].

As with DMS, the required sample volume for VAMS is in the order of microliters. However, VAMS devices allow the collection of a sample in a consistent volume, regardless of its viscosity, since they collect a fixed volume of sample [3,21]. Thus, the homogeneity of the sample is guaranteed, even in cases of higher viscosity of the matrix, since the collected volume is precise [22]. VAMS devices allow for the collection of fixed sample volumes, typically 10, 20, or 30 µL, which contributes to the accuracy and reproducibility of analytical results [17]. Additionally, they exhibit high precision in sample collection, with standard deviations of less than 0.4 µL for 10 µL blood samples [23]. To ensure the quality of the collected samples, it is essential that sampling procedures are correctly followed, including checking the collected sample volume and assessing sample integrity. The proper training of operators and the implementation of stringent protocols are crucial to minimize errors and ensure the consistency of the sample volumes [24]. VAMS devices also make it possible to overcome issues related to spot extractability, since it is not necessary to punch and cut the card as with DMS, and the entire tip containing the sample is analyzed [3,17]. In contrast to DMS, VAMS does not require a centrifugation step before extraction, making it simpler [17]. The sample homogeneity is of great importance for quantification in dry matrix [3]. However, when using DMS, the analyte is often retained in the center or on the periphery of the spot, depending on the affinity for the card, which makes the analysis difficult [3]. Also, the spread of blood on the card is a concern, and depending on the type of card used, different volumes of sample may be required to generate an identically sized patch [17,25]. Differences in scattering speed also affect analyte concentration and may even interfere with drying time [3,17]. For VAMS, this is not a concern as the device absorbs a fixed volume of sample [3,13]. Additionally, VAMS also presents advantages over DMS regarding contamination [3,17]. The VAMS device has a cartridge protector that seals the sample unlike DMS which, due to its design, leaves the dry spot exposed [17]. Despite the advantages that VAMS has, the costs associated with its use are much higher compared to the use of DMS [13]. Moreover, the VAMS device underfilling is also difficult to detect [13]. The relatively high cost of VAMS devices, primarily due to their single-use nature and specialized materials, can hinder adoption in resource-limited environments [13]. While VAMS offers significant benefits such as non-invasive sampling and stable transport, its affordability remains a barrier, especially in clinical settings with constrained budgets. Cost-reduction strategies, such as scaling production or using alternative materials, could help make VAMS a more viable option in low-resource contexts. VAMS devices are often made from plastic, raising concerns about their environmental impact due to their single-use nature [9,10]. Exploring sustainable alternatives, such as biodegradable or recyclable materials, could reduce their ecological footprint. Additionally, optimizing packaging and transport logistics could further decrease the environmental burden associated with VAMS usage.

Despite the differences, both VAMS and DBS show improvements in the matrix effect, compared to conventional sampling procedures [17]. Due to the drying process, handling, storage, and transportation of the samples are also simplified [8,11]. VAMS devices have been designed to facilitate direct application to the matrix to be sampled, without any kind of preliminary device treatment [26]. It is possible to pre-soak VAMS tips in additive solutions, such as anticoagulants or antioxidants, to prevent early coagulation on the tip or the oxidation and degradation of the biological sample [8]. Moreover, the addition of internal standard (IS) to real samples is nearly unfeasible due to the direct sample collection, so most ISs are added directly to the extraction solvent [8].

While the clinical and forensic applications of VAMS share core principles—such as the need for accurate and minimally invasive sampling—the specific requirements and challenges in these fields differ significantly. In clinical toxicology, VAMS is often used to monitor therapeutic drugs [27], detect disease biomarkers [28,29,30], or measure endogenous compounds [31,32], with a focus on patient compliance and ease of sample collection. In contrast, forensic toxicology prioritizes the detection of illicit substances [33,34,35,36,37], doping agents [31,38,39], and poisons, often under strict legal and procedural constraints. Despite these differences, both fields benefit from VAMS’s ability to standardize sample volumes, minimize environmental contamination, and ensure sample stability during transport and storage [3,10]. Highlighting these shared advantages, while recognizing the unique demands of each application, underscores the versatility of VAMS as a microsampling technique.

## 2. Volumetric Absorptive Microsampling in Clinical Toxicology

This section addresses the methods developed with VAMS used in the preparation and treatment of biological specimens for application in the clinic, for which the data obtained are shown and a critical discussion on their applications is made. The required literature search was performed using PubMed and ISI Web of Knowledge databases, and the search string was “VAMS and toxicology” or “volumetric absorptive microsampling and toxicology”. All articles were selected by three of the authors independently, to determine their importance in the context of the present review, and only those chosen by at least two of the authors were included in this review. Due to recent revisions, articles on the determination of antiepileptics, immunosuppressants, and disease biomarkers and on therapeutic drug monitoring were not considered for analysis and compilation.

VAMS has been applied for the extraction of various drugs and metabolites, endogenous substances, and chemical elements in biological specimens, and Table 2 summarizes the bioanalytical procedures published from 2020 to the present year that use the VAMS extraction technique with clinical applications.

Regarding antibiotics, Moorthy et al. [42] developed a methodology for the determination of cefepime in 10 µL of whole blood and its analysis by liquid chromatography coupled with tandem mass spectrometry (LC-MS/MS), for which they obtained a limit of quantification (LOQ) value of 100 ng/mL and recoveries between approximately 41 and 62%. The antibiotic was shown to be stable in dried samples for 3 months at −78 °C, and the method was successfully applied to whole blood microsamples in a pediatric clinical trial. The authors concluded that further studies are needed to evaluate the effect of hematocrit levels in clinical samples, but also that this method is an alternative sampling strategy for collecting samples from neonates with heel pricks and from children and adults with finger pricks. Although this technique was initially considered for the extraction of blood samples, it was also applied for the treatment of other biological samples. In the same year, Penot et al. [40] established a method for the quantification of doxycycline in only 10 µL of urine samples and its determination by the same analytical methodology implemented in the previously mentioned research. For this antibiotic, an LOQ value of 250 ng/mL was achieved, with an average recovery of 109% and stability for 31 days at room temperature and at −80 °C for 12 months. The authors concluded that this is an excellent alternative tool for monitoring the compliance of this antibiotic, even for large-scale populations, and as a transport and storage device.

The VAMS devices can also be implemented in the context of metal monitoring for clinical purposes. Capiau et al. [44] developed a method for the determination of cobalt in 10 µL of blood samples from patients with metal-on-metal prostheses, with analysis by inductively coupled plasma and detection by mass spectrometry, and for which, they obtained an LOQ value of 2 ng/mL. The authors believe that this technique is less affected by the hematocrit effect and that it represents an easy sampling that allows the patient to collect a capillary blood sample derived from a finger prick at home. Koutsimpani-Wagner et al. [54], on the other hand, developed a method for the biomonitoring of mercury in whole blood samples by direct mercury analysis, which may be suitable as an alternative sampling to determine its exposure by the general population, being stable for at least 4 weeks in the studied conditions.

In 2021, a methodology was developed by Delahaye et al. [46] for the determination of paracetamol (acetaminophen) and four of its metabolites in 10 µL samples of whole blood and analysis by LC-MS/MS. The authors achieved LOQ values between 10 and 100 ng/mL and obtained recoveries between 62 and 106% for the various compounds under study, and the longest stability achieved (3 months) was at −20 °C. The method was fully validated and showed that it can be used for pharmacokinetic studies to investigate paracetamol metabolism in different groups of patients. In the following year, the same working group implemented the previously described methodology for a pharmacokinetic study of these compounds in obese and non-obese patients, with the aim of establishing optimized dosing schemes for obese patients, given the prevalence of obesity. In this study, the differences between capillary (finger prick) and venous VAMS samples were studied, and it was concluded that the determination of these compounds under study in dried capillary blood samples could be used as an alternative matrix to conventional venous sampling in the investigation of paracetamol pharmacokinetics and may also serve as a basis for other pharmacokinetic studies [47].

Another application for the VAMS device is the study carried out by Jacobs et al. [49] for the quantification of 13 frequently prescribed antipsychotics (amisulpride, aripiprazole, clozapine, cyamemazine, haloperidol, melperone, olanzapine, paliperidone, pipamperone, promethazine, prothipendyl, quetiapine, and risperidone) in only 10 µL of whole blood and analysis by liquid chromatography coupled with high-resolution tandem mass spectrometry (LC-HRMS/MS). Although stability was only proven for just over half of the analytes under study, with a maximum of 2 weeks at 24 °C, the authors concluded that this dried matrix sampling was able to overcome the hematocrit problem in most cases and proved to be a promising alternative method for monitoring the adherence of these antipsychotics.

This same working group developed two more methodologies for the quantification of antihypertensive drugs with similar sample preparation techniques, using samples of 10 µL of finger-prick blood and also conducting analysis by LC-HRMS/MS. For the first study, they developed a strategy for monitoring the adherence of seven frequently prescribed antihypertensive drugs for which they obtained LOQ values that varied between 0.1 and 100 ng/mL, although for amlodipine neither the expected minimum concentration nor the therapeutic range was covered by the method, and recovery values were between 49 and 100%. The authors, in addition to the quantification of blood in VAMS, also quantified these same drugs in corresponding plasma samples, and the results demonstrated that the concentrations cannot be used interchangeably between the two biological samples, and for this reason, intervals of specific reference should be established. However, this new application was implemented for the investigated compounds, representing a sampling strategy with the advantage of stability in the dried matrix for all analytes except for lercanidipine [50]. More recently, they developed a methodology to quantify seven other antihypertensive drugs and metabolites (canrenone, enalaprilat, furosemide, hydrochlorothiazide, lisinopril, ramiprilat, and torasemide), for which they obtained LOQ values between 3 and 30 ng/mL and recoveries ranging from 68 to 110%. Also, in this study, a proof of concept was created with the objective of correlating the concentrations obtained between the corresponding finger-prick blood and plasma samples, for which they inferred the same conclusions as in the study described above. The authors also concluded that the analytes showed sufficient stability in dried matrix with VAMS and that this method allows a sampling easily accepted by the patient and with the possibility of being performed at home [56].

Also, noteworthy is the study carried out by Tagwerker et al. [51], who established a methodology for the analysis of 30 illicit compounds and 230 medications (analgesics/opiates, benzodiazepines, antipsychotics, antidepressants, depressants, anticonvulsants, muscle relaxants, stimulants, decongestants, appetite stimulants, antidotes, barbiturates, anti-inflammatory/NSAIDs, anti-histamines, cardiovascular agents, antimicrobials, gastrointestinal drugs, anti-emetics, antidiabetics, diuretics, phosphodiesterase inhibitors, corticosteroids/hormone therapies, antineoplastics/cancer therapies, and anti-dementia drugs (Parkinson’s/Alzheimer’s disease) and 43 confirmatory metabolites such as antidepressants, anti-inflammatory drugs, analgesics, anticonvulsants, and drugs for the treatment of diabetes in just 80 µL of blood and urine biological samples. In the analysis by high-performance liquid chromatography coupled with tandem mass spectrometry, the authors obtained values of limit of detection and LOQ between 0.5 and 125 ng/mL and stability up to 8 weeks at room temperature. This investigation concluded that this microsampling can be a reliable alternative for urine samples prone to adulteration, being an efficient method applicable to the determination of patient compliance, which offers low rejection rates, improves patient comfort, does not require transport in the cold chain, and increases operational efficiencies.

## 3. Volumetric Absorptive Microsampling in Forensic Toxicology

Just as the applications of VAMS in clinical settings were explored, its use in forensic scenarios has also been compiled and discussed. This review was conducted by searching the keywords “VAMS drugs” and “doping volumetric absorptive microsampling” in the PubMed and ISI Web of Knowledge databases. The inclusion and exclusion criteria for articles followed those applied in the clinical approach, except for the time range, which in this case had no restrictions. Table 3 summarizes all bioanalytical procedures utilizing VAMS for forensic toxicology analysis.

Concerning the use of VAMS in forensic and anti-doping scenarios, hematic samples constitute most of the applications for which new analytical methods have been reported. This is in line with the VAMS initial-development advantage, which was to overcome issues associated with DBS.

Several authors applied VAMS in a forensic context and presented noteworthy data that confirmed VAMS is a much more suitable dried sample approach for a wide range of illicit substances [74]. Nonetheless, it is important to mention that, thus far, its reported applicability in forensics is much lower than in the clinical context. When comparing VAMS with DBS, it is recognized that the difference in VAMS support’s nature and that of the DBS support (synthetic polymer vs. natural cellulose) may have an impact on the stability and extraction of the target analytes [36]. Marchand et al. [39] compared DBS with the VAMS approach for the determination of insulin-like growth factor 1 (IGF-1) in blood samples. For anti-doping purposes, the peptide hormone IGF-I is of special relevance [69]. This comparison proved that VAMS results were systematically better than those obtained with DBS when 20 µL VAMS was extracted with 150 µL of buffer and 40 µL DBS was extracted with 300 µL of buffer [39]. Also, Chang et al. [38], with the aim to determine anabolic steroids in dried blood samples, verified that the VAMS approach yielded much higher recoveries than those with the DBS approach, since the punched spots could result in a significant loss of approximately 20%. Protti et al. [73] evaluated VAMS suitability to determine natural and synthetic cannabinoids in whole blood and compared it to a DBS procedure. Overall, the compound levels found in DBS were always in good agreement with those found in VAMS. Nonetheless, the authors highlight that the obtained concentrations for DBS were accurate when the whole spot was analyzed [73].

Arguments are also reported concerning fluid blood samples and dried blood samples, and a careful comparison between the two should be made. This comparison is even more crucial when serum concentrations are the ones adopted and validated for anti-doping control.

Mongongu et al. [69] evaluated the hematocrit influence on IGF-I measurement by VAMS sampling, considering VAMS was created to collect blood in a precise amount. The authors reported that IGF-I can be quantified using VAMS with dried EDTA–human blood samples at physiological hematocrit levels (30–50%) using a single-point plasma sample calibrator made from human blood. In addition, the authors compared capillary blood samples collected with a TAP™ device and collected with VAMS, observing a bias of −46% in the concentration of IGF-I measured in dried capillary blood compared with that in the serum. The fact that heparin is present as an anticoagulant in the Tap™ device used to collect capillary blood can partially explain the obtained result. Moreover, apart from the matrix difference bias, it was concluded that, in a doping control scenario, VAMS is athlete-friendly and less invasive than traditional blood sampling [69]. Also, Marchand et al. [39], with the same aim, reported the need to perform a direct comparison between concentrations obtained for IGF-1 with VAMS and serum, since the latter is the only authorized sample for IGF-1 determination in anti-doping controls. The authors report that IGF-1 concentrations obtained from dried blood on VAMS were in accordance with those obtained in serum, although not completely similar [39]. Overall, there was an underestimation of IGF-1 concentrations with VAMS, which was supported by the lack of equivalence between 20 μL of serum and 20 µL whole blood [39].

Additionally, differences were also observed in Marchand et al. [39] study that compared venous DBS (venous blood + EDTA) and finger-prick DBS (capillary blood) when applied to VAMS. Al-Qurain et al. [65] clearly discussed the variability factors between these two samples since capillary blood (obtained from finger prick) is a combination of tissue and both venous and arterial blood. Variability might also be caused by lower supply of blood in finger extremities, leading to different concentrations when blood is taken from different locations, as well as due to the drug’s protein-binding effect [65]. Nevertheless, Al-Qurain et al. [65] compared a range of opioid concentrations in samples collected via VAMS with those prepared from whole blood and found that both inter- and intra-day variability were acceptable. Mandrioli et al. [36], with the aim to determine cocaine and its metabolites, used VAMS for both blood and plasma samples and also compared the results with those obtained for the fluid specimens. The hematocrit effect for these compounds is acknowledged to be very small, and this study confirmed that dried VAMS produced reliable data and closely resembled blood or plasma behavior [36]. Also, a consistency was reported between results obtained from capillary blood sampled by VAMS and venous blood obtained by venipuncture [36]. The authors notice that plasma-VAMS may have a slightly superior performance than blood-VAMS due to the absence of particle matter and, consequently, the hematocrit impact. However, since blood-VAMS may be acquired with a fingerpick and does not require any sample processing before sampling, it is consensually more practical than plasma-VAMS [36]. Protti et al. [73] reported a minimal effect of hematocrit on the measured cannabinoid levels in DBS. Additionally, a good correlation and agreement was observed between capillary-dried samples and venous-dried samples for both DBS and VAMS approaches [73].

In another study, Moorthy et al. [71] compared blood-VAMS to plasma-VAMS when aiming at determining cannabinoids. These authors observed lower concentrations of cannabinoids in whole blood when compared to plasma, and partitioning ratios of blood to plasma were adopted for pharmacokinetic studies. Still, concerning serum-sample-extrapolation problem, Chang et al. [38] observed no great discrepancies when comparing serum samples and VAMS dried blood concentrations of testosterone, assuring VAMS suitability for the purpose. However, the authors aimed at the determination of nine anabolic steroids using VAMS, but the serum and dried blood comparison was only reported and discussed for testosterone.

The extraction of the illicit substances from the VAMS dried tip was usually practical and simple, typically carried out with a mixture of organic solvents under agitation for a period. Nonetheless, some authors add a further extraction or cleanup procedure after the VAMS protocol. Although a few microliters of blood samples are dried in the VAMS tip, one should always consider that blood matrices are complex, and a simple and raw extraction with a solvent (e.g., methanol) elutes the target analytes but also matrix interferents. Nevertheless, when using an additional step of extraction and to improve selectivity, the analytes’ recovery might be affected.

Mestad et al. [35] used Parallel Artificial Liquid Membrane Extraction (PALME). This approach is justified by the large number of target analytes to be determined, including both polar and hydrophobic compounds. In these multi-target approaches, it is difficult to find a suitable extraction condition for all substances, and a single-step extraction from the tip could result in low recoveries for some of them. With the adopted procedure, Mestad et al. [35] reported recoveries greater than 70% for 13 out of the 17 target substances. Codeine and amphetamine, being two of the more polar compounds, had recoveries between 53 and 58%.

Protti et al. [66] coupled VAMS to microextraction by stop-and-go extraction (StAGE) tips for an enantioselective determination of clenbuterol. The authors highlight that the coupling of StAGE is very appropriate for the cleansing of small-volume dried specimens, obtaining satisfactory extraction yields in the 87 to 90% range.

Also, Mandrioli et al. [36] applied a dispersive pipette extraction (DPX) after VAMS tip extraction for further cleanup of the extract. The aim of the authors was to apply VAMS for forensic and anti-doping testing of cocaine and its metabolites in blood samples. The additional pre-concentration step with DPX only added one more minute to the entire sample treatment protocol, and good extraction yields were obtained (>85%) [36].

Moorthy et al. [71] subjected the blood-VAMS extracts to a solid-phase extraction procedure (SPE) for further cleanup and concentration of cannabinoids. The SPE was carried out with Oasis^®^ PRiME HLB µElution Plate, which resulted in overall recoveries ranging between 31 and 54%. These recoveries are lower than those obtained by Pigliasco et al. [34] for the same compounds. Although different extraction procedures were adopted by the different authors, the use of an additional cleanup step by Moorthy et al. [71] might justify some analyte loss and lower recoveries.

Several VAMS tips are commercially available, but the most-used tips were 20 μL tips. These are mainly chosen over the 10 μL tips to provide more material and increase the sensitivity of the analytical method. It is important to consider that the dried microsampling approach’s main drawback is the decrease in sensitivity when compared to sampling on a macroscopic scale. This can be partially compensated by direct MSn analysis [62]. Additionally, VAMS’s limited sample availability is also pointed out as a drawback, limiting the assortment of assays that can be run in a single microsample. This restriction can also be compensated by the usage of numerous VAMS techniques for each sampling session [66]. The latter can increase the overall quantification and detection limits of the method.

Although VAMS was initially developed for blood collection, there is a growing interest in its suitability for other biological specimens. Urine is the second-most-applied sample to VAMS in a forensic scenario. Great interest has been garnered by urine samples applied to VAMS for doping control, with dried urine spots (DUS) and VAMS becoming the most promising tools to improve the control strategies of illicit substances [62]. As in the case of blood, the inhomogeneity of urine sampled with VAMS is meant to be kept at a minimum or insignificant level, in contrast to what occurs for DUS [62].

Protti et al. [62] confirmed that a significantly lower stability is observed for fluid urine when compared to that of the dried matrix, and although small differences were reported between DUS and VAMS, greater mean stability was observed when VAMS was used. Greater stability was also reported for clenbuterol in urine samples by Protti et al. [66]. After 1 and 3 months, the authors observed that VAMS stored at room temperature provided greater recoveries than urine samples stored at −80 °C, and this stability improvement is partially attributed to the water loss in VAMS that can either slow down or stop most chemical enzymatic reactions [66].

Concerning glucocorticoids’ determination in urine samples, VAMS and DUS stored at room temperature for 3 months appeared to provide greater stability than in fluid urine, even when the latter is stored at −20°C or −80°C. VAMS also results in a greater apparent stability at room temperature, when compared with DUS, but no significant differences were found [68].

In another study by Protti et al. [31], VAMS was compared to DUS with the aim to determine 13 anabolic androgenic steroids in urine. In general, the authors again found better recoveries and precision when VAMS was adopted. Additionally, VAMS provided higher compound stability with yields greater than 88% and 82% after 3 months and 1 year at room temperature, respectively. The DUS stability was systematically lower with extraction yields greater than 85% after 3 months and 70% after 1 year at room temperature [31]. The authors also decided to compare fluid urine with both microsampling methods and found negligible differences between intercepts and regression coefficients. Protti et al. [72] also developed an analytical method to determine oxycodone and its major metabolites in urine samples using the VAMS approach. Using solely 10 µL, it was possible to achieve limits of quantification of 0.5 ng/mL and recoveries ranging from 76 to 88%. In this study, no clear winner was declared between the use of DUS and VAMS, although the latter was reported to lead due to slightly better precision and accuracy, apart from being less labor-intensive.

Oral fluid, being one of the most important biological specimens for the detection of recent illicit substance use, has also been sampled with VAMS. This sample can be obtained in a non-invasive manner, and a reasonable correlation with drug blood concentrations can be observed [75]. VAMS’s application to oral fluid has been studied to a lesser extent than urine, and up to date, it has only been used for drugs of abuse testing.

Morato et al. [70] developed a multimethod where VAMS was applied to collect oral fluid and allowed the simultaneous identification of 30 commonly used illicit substances. In this study, VAMS is pointed as a great alternative to touch spray (TS) swabs, which are frequently used in clinical settings but cannot collect a specific volume of material. Therefore, the quantitative potential of TS swabs was limited [70]. The authors describe a very promising approach requiring only 10 µL of oral fluid and reaching limit of detection below 5 ng/mL for most target compounds [70]. The coupling of this VAMS sampling with touch spray (TS) MS technique makes it even more interesting since it allows in situ immediate analysis [70].

When comparing the clinical and forensic applications of VAMS, both fields exhibit their precision in sample volume collection and the simplicity of their workflow. However, the limitations and benefits of VAMS manifest differently depending on the context. In clinical toxicology, the technique enhances therapeutic drug monitoring and patient adherence by enabling self-sampling and eliminating the need for cold chain logistics. This makes it particularly useful in remote or resource-limited settings. Meanwhile, in forensic toxicology, VAMS offers distinct advantages for fieldwork and post-mortem analysis by facilitating the detection of a wide range of substances with minimal degradation during transport.

Nonetheless, forensic applications often demand higher sensitivity and robustness due to legal requirements, as even minor inconsistencies in sample integrity could undermine case outcomes. Conversely, clinical settings may tolerate slight variations if they do not significantly impact patient care decisions.

In clinical settings, VAMS is employed for pharmacokinetic and pharmacodynamic studies, therapeutic drug monitoring, and disease management. It facilitates the precise tracking of drug levels and chronic condition monitoring, including diabetes, cardiovascular diseases, and infections. VAMS can be integrated into remote patient care and telemedicine, enabling patients to collect samples at home for efficient biomarker monitoring and genetic testing. It is a versatile tool applied in genomic diagnostics and precision medicine, offering innovative solutions for non-invasive prenatal testing and genetic screening.

In forensic science, VAMS is effectively utilized for toxicology analysis, drug and alcohol testing, and DNA collection in criminal investigations. It plays a pivotal role in postmortem toxicology, providing small yet accurate blood samples to detect drugs, alcohol, and poisons, thereby supporting law enforcement efforts. In cases of sexual assault, VAMS ensures discreet and high-quality evidence collection. It is also employed for monitoring individuals under parole or probation, reducing the need for invasive procedures.

VAMS technology is designed to maintain sample integrity, uphold the chain of custody, and comply with regulatory standards across both clinical and forensic applications.

Both contexts share challenges related to high costs and environmental concerns associated with the single-use plastic nature of VAMS devices. Addressing these limitations through technological innovations and the adoption of sustainable materials could further broaden its applicability, ensuring that VAMS remains a transformative tool in both clinical and forensic toxicology.

## 4. Conclusions

VAMS has proven to be a promising and reliable technique for the collection of biological samples in both clinical and forensic toxicology. Its ability to collect precise sample volumes with minimal invasiveness, combined with its feasibility of transport and storage at room temperature, positions it as an attractive alternative to traditional techniques and other dried matrix microsampling methods.

In clinical settings, VAMS has shown effectiveness in the quantitative and qualitative analyses of drugs, endogenous substances, and chemical elements in various biological matrices. It addresses limitations associated with the hematocrit effect and ensures greater sample homogeneity. In forensic toxicology, VAMS has expanded its applications to include the detection of illicit substances and doping control, demonstrating advantages in stability and consistency compared to conventional methods.

However, despite its benefits, VAMS is not without challenges. The cost of the devices, their single-use nature, and their plastic make remain significant concerns. It will be desirable in the future for devices to be developed where all or part of them are made from biodegradable materials. Issues such as the underfilling of samples and the limited volume available for multiple analyses highlight areas requiring technological and methodological improvements.

The ongoing development of VAMS, alongside advancements in sustainable materials and analytical techniques, is essential to expand its use and enhance its efficiency and environmental sustainability. Therefore, VAMS holds significant potential for revolutionizing analytical practices in toxicology, both in clinical and forensic contexts, particularly in resource-limited environments.

## Figures and Tables

**Table 1:** Bioanalytical microsampling techniques.

Microsampling Technique	Principal Practical Use	Advantages	Limitations
Volumetric adsorptive microsampling (VAMS)	Protein and genetic analysis Therapeutic drug monitoring (TDM) Biomarkers Clinical and forensic toxicology	Absorbs a fixed sample volume (less than ±5% of variations in volume) Hematocrit can potentially be ignored Comes in a protective casing, preventing contamination Easy and fast sample collection, storage, and transportation Can be used for self-sampling Improved stability	More expensive than DMS Requires drying
Dried matrix spots (DMS)	Protein and genetic analysis Therapeutic drug monitoring (TDM) Biomarkers Clinical and forensic toxicology	Small sample volume Low cost Improved sample stability due to the reduced water content Ease of use	Hematocrit effect in dried blood spots Variable spot size Requires drying Easily contaminated Higher matrix effects due to the hematocrit
Plasma extraction cards	Protein and genetic analysis Therapeutic drug monitoring (TDM)	Collection of plasma without the need for centrifugation	Hematocrit effect might be present Variable spot size Requires drying Easily contaminated
Microneedle	Clinical research Biomarker analysis	Capable of penetrating the skin and sampling at the same time Can be painless both during insertion and after removal	Expensive Requires special devices

**Table 2:** Bioanalytical procedures using VAMS approach for clinical applications.

Compounds	Sample (Volume, µL)	VAMS Procedure	Analytical Instrumentation	LOD; LOQ (ng/mL)	Recoveries (%)	Long-Term Stability	Reference and Year
Doxycycline	Urine (10)	Absorb: 5 s Dry: 2.5 h at room temperature Extract: 1.5 mL microtube with 500 μL of the mixture of water/acetonitrile/trifluoroacetic acid/acetic acid (85:15:0.1:0.5, *v*/*v*/*v*/*v*); the tube was vortex-mixed for 30 min at room temperature and protected from light and centrifuged for 10 min (25,000× *g*).	LC-MS/MS	166; 250	109	12 months at −80 °C; 31 days at room temperature	[40] 2020
Gefapixant	Whole blood (10)	Absorb: n.s. Dry: n.s. Extract: 200 µL of an extraction solution of acetonitrile/water/formic acid (80:20:0.1, *v*/*v*/*v*) along with 2.5 ng of SIL-IS, all vortexed for 1 h; a 50 µL aliquot of the sample extract was diluted with 200 µL of 0.1% formic acid.	LC-MS/MS	n.s.; 10	≥96	11 days at −20 °C; 133 days at room temperature; 11 days at 40 °C	[41] 2020
Cefepime	Whole blood (10)	Absorb: 2 s, 45° angle Dry: 1 h at room temperature Extract: A 50 μL volume of water was added to all samples, vortexed for 2 min (1000 rpm), placed in the incubator for 10 min at 37 °C, sonicated for 15 min, vortexed for 10 min (700 rpm), and centrifuged for 30 min (3220× *g*) at 4 °C.	LC-MS/MS	n.s.; 100	40.8–62.1	14, 28, and 91 days at −78 °C; 39 days at −20 °C; 7 days at 4 °C	[42] 2020
γ-hydroxybutyric acid	Whole blood (20)	Absorb: 2 to 4 s Dry: 1.5 h at room temperature Extract: A 200 µL volume of 0.1% formic acid in water was added, vortexed for 1 min, and sonicated for 15 min at 60 °C. Protein precipitation was carried out using 600 µL of acetonitrile, and the sample was vortexed for 30 s and centrifuged for 10 min (1700× *g*) at 4 °C. The supernatants were filtered.	UHPLC-MS/MS	250; 500	77–83	14 and 30 days at 21 °C (results n.s.)	[43] 2020
Cobalt	Whole blood (10)	Absorb: n.s. Dry: 2 h at room temperature Extract: A 1990 μL volume of a mixture containing 3 mM EDTA, 7.5% *v*/*v* butanol, 0.14 mM NH_4_OH, 1% *w*/*v* Triton X-100 and 0.1 μg/L Y in Milli-Q water was taken. Samples were shaken for 15 min (1000 rpm) at 60 °C.	ICP-MS	n.s.; 2	n.s.	48 days at room temperature; 48 h at 60 °C (results n.s.)	[44] 2020
Paracetamol–protein adducts (APAP-Cys^prot)^	(a) Plasma (10.) (b) Whole Blood (10)	Absorb: 2 s Dry: 2 h at room temperature Extract: Added 500 μL extraction solvent (methanol/water/formic acid; 80:20:0.01, *v*/*v*). Digestion: 100 μL of pronase solution and 100 μL of AMBIC (50 mM). 400 μL of methanol. Samples were shaken for 10 min (1400 rpm) at 23 °C and centrifuged (10 min, 10,000× *g*). A 500 μL aliquot of supernatant was evaporated and reconstituted with 65 or 100 μL of AMBIC (50 mM) for plasma samples and blood samples, respectively.	UHPLC-MS/MS	(a) n.s.; 0.05 µM (b) n.s.; 0.25 µM	(a) 95.5–97.7 (b) was evaluated across different hematocrit (Hct) levels, with no significant differences observed	6 months at −20 °C, 4 °C, and room temperature	[45] 2020
(a) Acetaminophen (b) Acetaminophen-Glucuronide (c) Acetaminophen-Sulfate (d) Acetaminophen-Mercapturate (e) Acetaminophen-Cysteine	Whole Blood (10)	Absorb: 2 s Dry: 2 h at room temperature Extract: A 500 μL volume of acetonitrile/water (80:20, *v*/*v*) extraction solvent was vortexed for 30 min (1400 rpm) at 60 °C and centrifuged for 10 min (10,000× *g*). A 450 μL aliquot of evaporated supernatant was reconstituted in 100 μL of water/formic acid (100:0.01, *v*/*v*).	LC-MS/MS	(a) n.s.; 100 (b) n.s.; 100 (c) n.s.; 100 (d) n.s.; 10 (e) n.s.; 10	(a) 79–82 (b) 87–101 (c) 92–106 (d) 73–78 (e) 62–64	3 months at −20 °C; 1 month at 4 °C; 3 days at room temperature; 1 week at 60 °C (for all the analytes)	[46] 2021
							[47] 2022
Hydroxychloroquine	Spiked blood (30)	Absorb: n.s. Dry: 2 h at room temperature Extract: A 500 μL volume of 1% ammonia solution was added to the microtube, vortex-mixed for 15 s, and sonicated for 5 min. A 500 μL volume of n-hexane/ethyl acetate (50: 50, *v*/*v*) was added to the tubes, which was vortexed for 15 s and centrifuged for 5 min (10,000 rpm).	HPLC-DAD	n.s.; 2	88.93–90.33	30 days at room temperature	[48] 2021
(a) Amisulpride (b) Aripiprazole (c) Clozapine (d) Cyamemazine (e) Haloperidol (f) Melperone (g) Olanzapine (h) Paliperidone (i) Pipamperone (j) Promethazine (k) Prothipendyl (l) Quetiapine (m) Risperidone	Whole Blood (10)	Absorb: 2 s Dry: 3 h at room temperature Extract: A 90 μL volume of purified water was added into 2 mL reaction tubes. Samples were shaken for 15 min (1500 rpm) at 37 °C. A 200 μL volume of acetonitrile was added, shaken for 30 min (1500 rpm) at 24 °C, and centrifugated for 10 min (15,000× *g*) at −10 °C.	LC-HRMS/MS	(a) n.s.; 50 (b) n.s.; 50 (c) n.s.; 50 (d) n.s.; 0.5 (e) n.s.; 0.5 (f) n.s.; 5 (g) n.s.; 5 (h) n.s.; 5 (i) n.s.; 50 (j) n.s.; 5 (k) n.s.; 0.5 (l) n.s.; 50 (m) n.s.; 0.5	(a) 123–146 (b) 110–131 (c) 102–117 (d) 49–68 (e) 71–103 (f) 54–67 (g) 13–34 (h) 112–138 (i) 112–123 (j) 52–60 (k) 32–63 (l) 129–146 (m) 107–125	(a) 2 weeks at 24 °C (b) 2 weeks at 24 °C (c) 2 weeks at 24 °C (d) It was not stable (e) 1 week at 24 °C (f) It was not stable (g) It was not stable (h) 2 weeks at 24 °C (i) 2 weeks at 24 °C (j) It was not stable (k) It was not stable (l) 2 weeks at 24 °C (m) 2 weeks at 24 °C	[49] 2021
(a) Amlodipine (b) Bisoprolol (c) Candesartan (d) Carvedilol (e) Lercanidipine (f) Losartan-carboxylic acid (g) Metoprolol (h) Nebivolol (i) Telmisartan (j) Valsartan	Whole Blood (10)	Absorb: 2 s Dry: 3 h at room temperature Extract: A 90 µL volume of purified water was added into 2 mL reaction tubes. Samples were shaken for 15 min (1500 rpm) at 37 °C. A 200 µL volume of precipitation agent of methanol/acetonitrile (30:70, *v*/*v*) was added, shaken for 30 min (1500 rpm) at 24 °C, and centrifugated for 15 min (15,000× *g*) at −10 °C.	LC-HRMS/MS	(a) n.s.; 4 (b) n.s.; 1 (c) n.s.; 1 (d) n.s.; 4 (e) n.s.; 0.1 (f) n.s.; 25 (g) n.s.; 1 (h) n.s.; 0.1 (i) n.s.; 1 (j) n.s.; 100	(a) 53–72 (b) 82–100 (c) 78–90 (d) 69–99 (e) 49–77 (f) 69–90 (g) 73–86 (h) 70–73 (i) 67–87 (j) 69–89	(a) 2 weeks at 24 °C (b) 2 weeks at 24 °C (c) 2 weeks at 24 °C (d) 2 weeks at 24 °C (e) It was not stable (f) 2 weeks at 24 °C (g) 1 week at 24 °C (h) 2 weeks at 24 °C (i) 2 weeks at 24 °C (j) 2 weeks at 24 °C	[50] 2021
230 medications and 30 illicit compounds	Whole blood (4 × 20) and urine (4 × 20)	Absorb: n.s. Dry: n.s. Extract: Ninety-six-well plate containing 200 μL methanol-based solvents and VAMS tips were incubated for 15 min at room temperature and sonicated with circulating distilled water for 5 min at 4 °C. After the manual removal of the tip and stylus from each well, 200 μL of water was added.	LC-MS/MS	0.5–125; 0.5–125 (for both samples)	<75	1 month and 8 weeks at room temperature (for all the analytes)	[51] 2021
Rosuvastatin	Spiked blood (10)	Absorb: n.s. Dry: 1 h at room temperature Extract: A 250 μL volume of methanol or acetonitrile as an extraction solvent was added. The tubes were vortexed for 10 min (1000 rpm) at room temperature and sonicated for 10 min. A 200 μL aliquot of evaporated supernatant was reconstituted with 50 μL of 50% methanol.	LC-MS/MS	n.s.; 1	102.75–117.33	30 days at room temperature	[52] 2021
Thiamine	Venous whole blood (50)	Absorb: 6 s Dry: 2.5 h at ambient temperature Extract: A 150 μL volume of 12% TCA (containing 5 ng/mL D_3_TDP) as the extraction solvent. The tubes were shaken at 1000 rpm for 30 min at 60 °C and centrifuged for 5 min at 4 °C and 12,000× *g*. The supernatant (100 μL) was transferred to an amber glass vial.	LC-MS/MS	n.s.; 9.3	92–107	2 weeks and 1 month at −20 °C, 4 °C and room temperature	[53] 2021
Thiamine	Venous whole blood (10)		UHPLC-MS/MS		n.s.	“1 week at 60 °C or high humidity 1 month at ambient conditions”	[11] 2021
Mercury	Whole blood (20)	Absorb: n.s. Dry: 2 h at room temperature Extract: n.s.	DMA	0.18; 0.61 (for single tip); 0.10; 0.33 (for double tip)	70–130	1, 2, and 4 weeks at −20 °C, room temperature, and 40 °C	[54] 2022
Acetylsalicylic acid	Whole blood (10)	Absorb: 2 s Dry: 1 h at room temperature Extract: The tips were placed in tubes, and for protein precipitation, they were sonicated with 250 μL of acetonitrile for 15 min and centrifuged for 10 min (10,000 rpm) at 4 °C.	LC-MS/MS	n.s.; n.s.	87.7–101	1 month at 4 °C, room temperature, and −20 °C	[55] 2022
(a) Canrenone (b) Enalaprilat (c) Furosemide (d) Hydrochlorothiazide (e) Lisinopril (f) Ramiprilat (g) Torasemide	Whole blood (10)	Absorb: 2 s Dry: 3 h at room temperature Extract: A 90 µL volume of purified water was added into 2 mL reaction tubes. A 200 µL volume of the precipitation agent containing methanol/acetonitrile (30:70, *v*/*v*) was added, and the samples were shaken for 15 min (1500 rpm) at 37 °C. Samples were shaken for 30 min (1500 rpm) at room temperature and centrifugated for 15 min (15,000× *g*) at −10 °C.	LC-HRMS/MS	(a) n.s.; 30 (b) n.s.; 3 (c) n.s.; 5 (d) n.s.; 10 (e) n.s.; 5 (f) n.s.; 10 (g) n.s.; 3	(a) 68–92 (b) 80–101 (c) 86–110 (d) 82–110 (e) 71–97 (f) 73–98 (g) 77–102	2 weeks at 24 °C (for all the analytes)	[56] 2023
Favipiravir	Whole blood (n.s.)	Absorb: 2 s Dry: 2 h at room temperature Extract: A 500 μL volume of methanol was added to the microtube, vortexed for 30 s, sonicated for 15 min, and centrifuged for 10 min (10,000 rpm).	HPLC-DAD	n.s.; 500	69.39–73.30	28 days at −20 °C	[57] 2023
(a) Favipiravir (b) Remdesivir	Whole blood (10)	Absorb: 2 s Dry: 2 h at room temperature Extract: A 500 μL volume of methanol was vortexed for 30 s, sonicated for 20 min, and centrifuged for 10 min at 10,000 rpm. Supernatant was evaporated for 30 min at 40 °C.	LC-MS/MS	(a) n.s.; 500 (b) n.s.; 2	(a) 65.64–75.11 (b) 80.55–83.38	(a) 14 days at −20 °C (b) 7 days at −20 °C	[58] 2023
(a) 5-methyltetrahydrofolate (5MTHF) (b) MeFOX (c) 10-formylfolic acid (10FoFA (d) 5,10-methenyltetrahydrofolate (5,10CH + THF) (e) tetrahydrofolate (THF)	Whole Blood (10)	Absorb: 6 s, 45° angle Dry: 2.5 h at room temperature Extract: Mixing was performed for 90 min at 25 °C and 1200 rpm. A 165 μL volume of the extraction solvent (50 mM sodium phosphate buffer, pH 7.4, containing 1% ascorbic acid and 0.5% DL-dithiothreitol) was added. The extract was incubated in 35 μL charcoal-stripped rat serum for 1 h at 37 °C. A 160 μL volume of the extract was centrifuged twice for 15 min at 14,000× *g* and 4 °C.	LC-MS/MS	Limit of acceptance	Varies with temperature and stabilizers	Between 1 and 2 weeks	[59] 2024

DMA: direct mercury analysis; HPLC-DAD: high-performance liquid chromatography diode array detector; ICP-MS: inductively coupled plasma mass spectrometry; LC-HRMS/MS: liquid chromatography–high-resolution-tandem mass spectrometry; LC-MS/MS: liquid chromatography–tandem mass spectrometry; LOD: limit of detection; LOQ: limit of quantification; n.s.: not specified; UHPLC-MS/MS: ultra-high-performance liquid chromatography–tandem mass spectrometry.

**Table 3:** Bioanalytical procedures using VAMS approach for forensic toxicology application.

Compounds	Sample (Volume, µL)	VAMS Procedure	Analytical Instrumentation	LOD; LOQ (ng/mL)	Recoveries (%)	Long-Term Stability	Reference and Year
(a) Cannabidiol (CBD) (b) Δ9-Tetrahydrocannabinol (Δ9-THC) (c) 7-Carboxy-cannabidiol (CBD-COOH) (d) 7-Hydroxy-cannabidiol (CBD-OH) (e) Cannabinol (f) 11-Hydroxy-Δ9-tetrahydrocannabinol (THC-OH) (g) 11-Nor-9-carboxy-Δ9-tetrahydrocannabinol (THC-COOH)	Oral fluid (n.s.)	Absorb: 10 s Dry: 1 h at room temperature in the dark Extract: A 250 µL volume of dichloromethane was added and vortexed for 15 min at 1000 rpm. The supernatants were evaporated to dryness. Dried residues were reconstituted in 50 µL of methanol/water with 1% (*v*/*v*) formic acid (80/20%, *v*/*v*).	UHPLC-HR/MS	(a) 0.1; 0.5 (b) 0.1; 0.5 (c) 0.2; 0.5 (d) 0.1; 0.5 (e) 0.1; 0.5 (f) 0.1; 0.5 (g) 0.2; 0.5	n.s.	(a) 72 h in autosampler at 10 °C and 1 month at −20 °C (b) 72 h in autosampler at 10 °C and 1 month at −20 °C (c) 72 h in autosampler at 10 °C and 1 month at −20 °C (d) 72 h in autosampler at 10 °C (e) 72 h in autosampler at 10 °C and 1 month at −20 °C (f) 72 h in autosampler at 10 °C and 1 month at −20 °C (g) 72 h in autosampler at 10 °C and 1 month at −20 °C	[60] 2024
90 legal and illegal drugs	Whole blood (20)	Absorb: 7 s Dry: 6 h at room temperature in the dark Extract: A 500 µL volume of the extraction solution (75% water with 1% FA, 10% methanol, 15% acetonitrile) was added, then sonicated for 30 min, and stirred at room temperature for another 30 min. Supernatants were recovered and evaporated to dryness under a nitrogen stream at 40 °C. The dried residues were reconstituted in 50 µL of the recovery solution (mixture (80/20%, *v*/*v*) of water/acetonitrile with 1% (*v*/*v*) FA).	LC-HRMS	1.25–500; n.s.	98	72 h at room temperature	[33] 2023
(a) Cannabidiol (CBD) (b) Δ9-Tetrahydrocannabinol (THC) (c) Cannabidiol-7-oic acid (7-COOH-CBD) (d) 7-Hydroxy-cannabidiol (7-OH-CBD) (e) 6-Alpha-hydroxy-cannabidiol (6-α-OH-CBD) (f) 6-Beta-hydroxycannabidiol (6-β-OH-CBD) (g) 11-Hydroxy-Δ9-tetrahydrocannabinol (11-OH-THC) (h) 11-Nor-9-carboxy-Δ9-tetrahydrocannabinol (THCCOOH)	Whole blood (30)	Absorb: n.s. Dry: n.s. Extract: (Basic extraction) 100 µL ammonium hydroxide (pH 9) after the addition of 4 mL hexane/ethyl acetate (9:1); horizontal agitation for 30 min; centrifugation for 3 min at 3500 rpm; transfer of supernatant; (acid extraction) 15 µL of formic acid (pH 3) after the addition of 4 mL hexane/ethyl acetate (9:1); vortex for 10 s, mix for 10 min and centrifuge at 5000× *g* for 5 min; combine and dry supernatants	UHPLC-MS/MS	(a) 0.50; 1.50 (b) 0.10; 0.25 (c) 1.10; 3.50 (d) 0.50; 1.50 (e) 0.10; 0.25 (f) 0.10; 0.25 (g) 0.10; 0.25 (h) 0.10; 0.25	(a) 63–69 (b) 80–89 (c) 96–98 (d) 92–98 (e) 78–97 (f) 82–90 (g) 78–86 (h) 84–94	(a–h) 1 month at room temperature	[34] (2022)
Continuous erythropoietin receptor activator (CERA)	Whole blood (20)	Absorb: n.s. Dry: 1 h at room temperature Extract: 1% Tween in 1 mL H_2_O was added and incubated for 15 min in a thermoshaker at 450 rpm and 37 °C; centrifuged briefly, sonicated for 15 min, and kept in a thermoshaker for another cycle of 15 min.	ELISA assay	n.s.	n.s.	n.s.	[61] (2022)
(a) Alprazolam (b) Amphetamine (c) Clonazepam (d) Cocaine (e) Codeine (f) Diazepam (g) Flunitrazepam (h) MDMA (i) Methadone (j) Methamphetamine (k) Morphine (l) Nitrazepam (m) Oxazepam (n) Oxycodone (o) Tramadol (p) Zolpidem (q) Zopiclone	Whole blood (20)	Absorb: 2 s Dry: 2 h at room temperature Extract: Parallel Artificial Liquid Membrane Extraction (PALME) with 185 µL ammonium carbonate buffer pH 9.3; a supported liquid membrane (SLM) was prepared with 4 µL of 6-undecanone/dihexyl ether (1:1) with 1% trioctylamine (*w*/*w*/*w*) onto polyvinylidene fluoride (PVDF) filters. Acceptor wells filled with 100 µL of DMSO were mixed with 200 mM HCOOH (75:25, *v*/*v*) and sonicated for 2 h. Then, 1 mL of acetonitrile was added, vortex-mixed for 10 min, and centrifuged at 12,000 rpm for 10 min.	UHPLC-MS/MS	(a) n.s.; 1 (b) n.s.; 5 (c) n.s.; 1 (d) n.s.; 1 (e) n.s.; 5 (f) n.s.; 1 (g) n.s.; 1 (h) n.s.; 5 (i) n.s.; 5 (j) n.s.; 5 (k) n.s.; 3 (l) n.s.; 1 (m) n.s.; 5 (n) n.s.; 5 (o) n.s.; 5 (p) n.s.; 1 (q) n.s.; 1	(a) 78–85 (b) 50–55 (c) 108–120 (d) 109–111 (e) 54–62 (f) 89–91 (g) 107–111 (h) 70–71 (i) 79–105 (j) 72–73 (k) 9–10 (l) 94–115 (m) 97–106 (n) 117–138 (o) 113–114 (p) 106–108 (q) 44–46	(a–q) 14 days at room temperature	[35] (2021)
(a) GHRP-1 (b) GHRP-2 (c) GHRP-6 (d) Hexarelin (e) Alexamorelin, (f) Triptorelin (g) AOD9604 (h) CJC-1293 (i) Desmopressin (j) TB-500 (k) hCG (l) ACTH (tetracosactide)	Urine (10)	Absorb: 5 s Dry: 1 h at room temperature Extract: 100 µL methanol, 10 min ultrasound-assisted extraction, plus 30 s vortex assisted extraction, plus 10 min ultrasound-assisted extraction.	LC-MS/MS	(a) 0.3; 1 (b) 0.3; 1 (c) 0.3; 1 (d) 0.3; 1 (e) 0.3; 1 (f) 0.3; 1 (g) 0.3; 1 (h) 0.1; 0.3 (i) 0.3; 1 (j) 0.3; 1 (k) 3; 10 (l) 3; 10	(a) 84–88 (b) 85–90 (c) 86–90 (d) 86–89 (e) 85–88 (f) 87–90 (g) 79–85 (h) 80–84 (i) 88–92 (j) 85–88 (k) 80–84 (l) 79–84	(a–l) 20 days at room temperature	[62] (2021)
Erythropoietin (EPO) transgene	Whole blood (20)	Absorb: n.s. Dry: overnight at room temperature Extract: 25 μL of Tris-HCl, 0.1 mM EDTA (TE), pH 9.0	qPCR	Cq values of 37 to 39; n.s.	n.s.	1 month at room temperature	[63] (2021)
Phosphatidylethanol (PEth)	Whole blood (10)	Absorb: 2 s Dry: 2 h at room temperature Extract: 250 µL of 2 mM ammonium acetate and 0.01% formic acid in a 2/8/0.2 water/isopropanol/formic acid mixture and 60 µL of methanol; shaking (1400 rpm) for 1 h	LC-MS/MS	1.7; 18.4	44–64	1 week at 4 °C 1 week at 45 °C 1 month at room temperature (EQA) Up to 400 days at room temperature	[64] (2021)
(a) Oxycodone (b) Tramadol (c) Fentanyl (d) Noroxycodone (e) Oxymorphone (f) O-desmethyltramadol (ODT) (g) N-desmethyltramadol (NDT) (h) Norfentanyl	Whole blood (20)	Absorb: 3 s Dry: 3 h at room temperature Extract: 120 µL methanol sonicated for 2 h; add 1 mL of acetonitrile, and vortex-mix for 10 min and centrifuge at 12,000 rpm for 10 min; supernatant is evaporated to dryness.	LC-MS/MS	(a) n.s.; 0.2 (b) n.s.; 10 (c) n.s.; 0.2 (d) n.s.; 0.2 (e) n.s.; 0.2 (f) n.s.; 10 (g) n.s.; 10 (h) n.s.; 0.2	(a) 99–104 (b) 96–107 (c) 96–105 (d) 86–92 (e) 93–105 (f) 81–102 (g) 104–110 (h) 95–103	(a–h) n.s.	[65] (2021)
Clenbuterol (CBT)	Urine (20)	Absorb: 5 s Dry: 1 h at room temperature Extract: 1 mL of methanol, sonicated for 20 min and evaporated to dryness.	LC-MS/MS	n.s.; 0.3	87–90	3 months at room temperature	[66] (2021)
Cannabidiol (CBD)	Venous blood (n.s.), capillary blood (n.s.)	Absorb: n.s. Dry: 1 h at room temperature Extract: 200 µL of methanol, sonicated 10 min, and centrifuged at 14,000 rpm for 10 min	LC-MS/MS	n.s.; 1	n.s.	7, 14, 21, and 28 days at −20 °C and 25 °C	[67] 2020
(a) Cortisol (CRL) (b) Corticosterone (CRT) (c) Cortisone (CRN) (d) Dexamethasone (DMT) (e) Methylprednisolone (MPN) (f) Fludrocortisone (FDC)	Urine (30)	Absorb: 5 s Dry: 1 h at room temperature Extract: Microwave-assisted extraction at 900 W for 20 s in 500 µL of methanol; then, ultrasound-assisted extraction for 5 min and vortex-mixing for 1 min	LC-MS/MS	(a) 4; 12 (b) 2; 7 (c) 1.5; 5 (d) 3.5; 10 (e) 0.4; 1.5 (f) 8; 25	(a) 87–91 (b) 91–99 (c) 86–88 (d) 82–86 (e) 88–99 (f) 92–94	(a–f) 3 months at room temperature	[68] (2020)
Insulin-like growth factor I (IGF-1)	Whole blood (20)	Absorb: 2 s, 45° angle Dry: 2 h at room temperature Extract: Transferred to 2 mL Protein LoBind^®^ tube containing 400 µL of saline solution, and then sonicated for 30 min. A 200 µL volume of acetonitrile containing 5% acetic acid was added and mixed at 900 rpm for 10 min; centrifuged at 14,000× *g* for 10 min; supernatant transferred to a new 2-mL Protein LoBind^®^ tube containing 400 µL of 5% NH_4_OH; vortexed and centrifuged at 14,000 × *g* for 10 min; supernatant submitted to mixed-mode strong-anion µSPE.	LC-HRMS	n.s; 25	89–95	n.s.	[69] (2020)
Insulin-like growth factor I (IGF-1)	Whole blood (20)	Absorb: n.s. Dry: 2 h at room temperature Extract: 1 h incubation on a rotating wheel at 20 rpm at room temperature in 150 µL saline buffer without antiproteases	Automated IGF-1 immunoassay	n.s.; 10	74–98	1 month at room temperature	[39] (2020)
(a) Nandrolone (b) Boldenone (c) Mesterolone (d) Testosterone (e) Drostanolone (f) Metenolone (g) Metandienone (h) Oxandrolone (i) DHCMT	Whole blood (20)	Absorb: n.s. Dry: 2 h at room temperature Extract: One milliliter of water and two milliliters of MTBE were added and kept in an ultrasonic bath for 15 min, rotary mixed for 15 min, and centrifuged at 3000 rpm for 5 min. The supernatant was transferred to another tube and dried under nitrogen at 60 °C for 10 min.	GC-MS/MS	(a) 0.20; 1.56 (b) 0.20; 1.56 (c) 0.20; 1.56 (d) 0.10; 0.78 (e) 0.20; 1.56 (f) 0.10; 1.56 (g) 0.10; 0.78 (h) 0.78; 1.56 (i) 0.78; 0.78	(a) 74 (b) 73 (c) 55 (d) 89 (e) 51 (f) 60 (g) 57 (h) 72 (i) 43	(a–i) 2 months at −20 °C (a–i) 2 months at 4 °C (a–i) 2 months at room temperature (a) 2 months at −20 °C better than at room temperature	[38] (2020)
(a) Cocaine (COC) (b) Benzoylecgonine (BEG) (c) Ecgonine methyl ester (EME) (d) Cocaethylene (CET)	Whole blood (20), plasma (20)	Absorb: 2 s, 45° angle Dry: 1 h at room temperature Extract: 100 µL methanol/acetonitrile/50 mM, pH 3.0, phosphate buffer (10/15/75, *v*/*v*/*v*); 20 min ultrasound-assisted extraction at 40 kHz; DPX pretreatment is required.	LC-MS/MS	(a) 0.6; 2 (b) 0.3; 1 (c) 0.8; 2.5 (d) 0.6; 2	(a) 91–98 (b) 88–94 (c) 87–95 (d) 86–94	(a–d) 2 months at room temperature	[36] (2020)
(a) Nandrolone (b) 1-Androstenedione (c) DHEA (d) Testosterone (e) Epitestosterone (f) DHT (g) Methandrostenolone (h) Norethandrolone (i) Mesterolone (j) Clostebol (k) Stanozolol (l) Fluoxymesterone (m) Danazol	Urine (30)	Absorb: 5 s Dry: air blowing 20 min at room temperature Extract: 500 µL methanol, and 5 min ultrasonication	LC-MS/MS	(a) 0.3; 1 (b) 0.5; 1.5 (c) 0.5; 1.5 (d) 0.3; 1 (e) 0.3; 1 (f) 0.5; 1.5 (g) 0.5; 1.5 (h) 0.5; 1.5 (i) 0.5; 1.5 (j) 0.5; 1.5 (k) 0.5; 1.5 (l) 0.5; 1.5 (m) 0.5; 1.5	(a) 81–82 (b) 83–86 (c) 90–92 (d) 77–80 (e) 79–82 (f) 80–83 (g) 83–86 (h) 77–79 (i) 80–81 (j) 82–84 (k) 83–87 (l) 81–84 (m) 81–85	(a–m) 3 months up to a year at room temperature	[31] (2020)
(a) 6-MAM (b) Alfentanil (c) Alprazolam (d) Amitriptyline (e) Amphetamine (f) Butylone (g) BZE (h) Clozapine (i) Cocaine (j) Codeine (k) Diazepam (l) Fentanyl (m) Furanylfentanyl (n) Haloperidol (o) Hydrocodone (p) Ketamine (q) MAMP (r) MBDB (s) MDEA (t) MDMA (u) Mephedrone (v) Methadone (w) Morphine (x) Naphyrone (y) Norcarfentanil (z) Oxycodone (µ) Paroxetine (α) Remifentanil (β) Sufentanil (γ) Venlafaxin	Oral fluid (10)	Absorb: 6 s Dry: 15 min using an electric vacuum desiccator Extract: Spray solvent with acetonitrile−isopropyl alcohol (1:1) mixture with formic acid 0.1% (*v*/*v*) octyl β-D-glucopyranoside; apply to the tip using a fused-silica capillary		(a) 4.86; 16.19 (b) 0.08; 0.28 (c) 0.49; 1.62 (d) 0.17; 0.55 (e) 11.28; 37.59 (f) 2.97; 9.89 (g) 1.25; 4.17 (h) 0.53; 1.78 (i) 0.39; 1.30 (j) 0.97; 3.22 (k) 1.15; 3.83 (l) 0.08; 0.25 (m) 0.08; 0.25 (n) 0.09; 0.30 (o) 0.22; 0.74 (p) 4.49; 14.97 (q) 24.49; 81.64 (r) 0.50; 1.67 (s) 0.44; 1.47 (t) 0.20; 0.67 (u) 15.39; 51.29 (v) 0.196; 0.655 (w) 3.24; 10.80 (x) 0.093; 0.309 (y) 0.12; 0.39 (z) 3.94; 13.13 (µ) 1.09; 3.62 (α) 0.45; 1.49 (β) 0.08; 0.25 (γ) 0.24; 0.79	(a) 88–96 (b) 96–113 (c) 112–143 (d) 79–90 (e) 85–115 (f) 65–96 (g) 88–121 (h) 91–94 (i) 85–114 (j) 94–110 (k) 61–71 (l) 87–88 (m) 64–122 (n) 80–102 (o) 91–115 (p) 89–98 (q) 88–103 (r) 73–106 (s) 88–96 (t) 89–100 (u) 35–283 (v) 94–94 (w) 59–76 (x) 95–96 (y) 111–146 (z) 84–89 (µ) 81–87 (α) 99–111 (β) 67–76 (γ) 102–130	(a–y) 1 week at −20 °C	[70] (2019)
(a) Delta-9-tetrahydrocannabinol (THC) (b) Cannabidiol (CBD) (c) Cannabinol (CBN)	Whole blood (20)	Absorb: 2 s, 45° angle Dry: 60 h at room temperature Extract: 40 µL of 0.1 M zinc sulfate–0.1 M ammonium acetate (50:50, *v*/*v*) and 250 µL of precipitation solution containing 0.1% formic acid in acetonitrile; vortexed for 15 min at 800 rpm, sonicated for 15 min, and centrifuged for 15 min at 4000 rpm at 4 °C; supernatant subjected to solid-phase extraction.	UHPLC-MS/MS	(a) n.s.; 1 (b) n.s.; 0.5 (c) n.s.; 0.5	(a) 36–54 (b) 31–40 (c) 37–43	(a–c) 2 weeks at 4 °C and room temperature (a–c) −20 °C for 1 month (a–c) −78 °C for 2 months	[71] (2019)
(a) Oxycodone (OXC) (b) Noroxycodone (NOC) (c) Oxymorphone (OMR)	Urine (10)	Absorb: 5 s Dry: 1 h at room temperature Extract: 100 µL methanol, 20 min ultrasound-assisted extraction, and evaporate to dryness	LC-MS/MS	(a) 0.2; 0.5 (b) 0.2; 0.5 (c) 0.2; 0.5	(a) 80–88 (b) 83–87 (c) 76–85	(a–c) 2 months at room temperature	[72] (2018)
(a) Δ9-Tetrahydrocannabinol (THC) (b) 11-Hydroxy-Δ9-tetrahydrocannabinol (THC–OH) (c) 11-Nor-9-carboxy-Δ9-tetrahydrocannabinol (THC–COOH) (d) JWH–018 (e) JWH-073 (f) JWH–250 (g) JWH–200 (h) HU–211 (i) CP 47,497 (j) JWH-019 (k) JWH-122 (l) JWH-081 (m) AM-2201	Whole blood (10)	Absorb: 2 to 4 s Dry: 1 h at room temperature Extract: 500 µL methanol, vortex-mix for 1 min; ultrasound agitation for 15 min; evaporate to dryness	LC-MS/MS	(a) 0.03; 0.1 (b) 0.05; 0.2 (c) 0.05; 0.2 (d) 0.05; 0.2 (e) 0.03; 0.1 (f) 0.05; 0.2 (g) 0.03; 0.1 (h) 0.1; 0.5 (i) 0.1; 0.5 (j) 0.03; 0.1 (k) 0.03; 0.1 (l) 0.03; 0.1 (m) 0.03; 0.1	(a) 93–97 (b) 85–91 (c) 84–93 (d) 87–95 (e) 89–97 (f) 85–95 (g) 88–94 (h) 81–91 (i) 81–90 (j) 85–93 (k) 86–93 (l) 88–94 (m) 83–92	(a–m) 30 days at room temperature	[73] (2017)
(a) Methylone (b) Ethyone (c) Butylone (d) Mephedrone (4-MMC)24 (e) 4-Methylethcathinone (4-MEC) (f) 3,4-Methylenedioxypyrovalerone (MDPV)	Urine (10), plasma (10), oral fluid (10)	Absorb: 2 to 4 s Dry: 1 h at room temperature Extract: 500 µL methanol, ultrasound agitation for 15 min, and vortex for 1 min	LC-MS/MS	(a) 3; 10 (b) 3; 10 (c) 3; 10 (d) 3; 10 (e) 3; 10 (f) 3; 10	(a) 79–89 (b) 80–88 (c) 81–86 (d) 76–81 (e) 75–76 (f) 77–77	(a–f) 7 days at room temperature	[37] (2016)

ELISA: enzyme-linked immunosorbent assay; GC-MS/MS: gas chromatography–tandem mass spectrometry; LC-HRMS: liquid chromatography–high-resolution mass spectrometry; LC-MS/MS: liquid chromatography–tandem mass spectrometry; LOD: limit of detection; LOQ: limit of quantification; n.s.: not specified; qPCR: real-time polymerase chain reaction; UHPLC-MS/MS: ultra-high-performance liquid chromatography–tandem mass spectrometry.

## Data Availability

This systematic search was performed via the databases of Web of Science and SCOPUS.
